# Can Galectin-3 Be Used as a Predictor of Obstructive Sleep Apnea Severity: Insights from High-Volume Patient Single Center

**DOI:** 10.3390/diagnostics15030375

**Published:** 2025-02-05

**Authors:** Milica Brajkovic, Sofija Nikolic, Viseslav Popadic, Natasa Milic, Nina Rajovic, Novica Nikolic, Ana Sekulic, Marija Brankovic, Mihailo Stjepanovic, Spasoje Popevic, Branko Milovanovic, Marija Zdravkovic

**Affiliations:** 1 Clinic for Internal Medicine, University Clinical Hospital Center Bezanijska kosa, 11000 Belgrade, Serbia; nikolic.sofija94@gmail.com (S.N.); viseslavpopadic@yahoo.com (V.P.); manive23@gmail.com (M.B.); 2 Faculty of Medicine, University of Belgrade, 11000 Belgrade, Serbia; silly_stat@yahoo.com (N.M.); nina94rajovic@gmail.com (N.R.); sekulic.ana@bkosa.edu.rs (A.S.); mihailostjepanovic@gmail.com (M.S.); spasapop@gmail.com (S.P.); 3 Department of Internal Medicine, Division of Nephrology and Hypertension, Mayo Clinic, Rochester, MN 55905, USA; 4 Department of Anesthesiology and Reanimation, University Clinical Hospital Center Bezanijska kosa, 11000 Belgrade, Serbia; novica.nikolic87@yahoo.com; 5 Clinic for Pulmonology, University Clinical Center of Serbia, 11000 Belgrade, Serbia; 6 Clinic for Cardiology, Military Medical Academy, 11000 Belgrade, Serbia; milovanovicbrankoo@gmail.com

**Keywords:** obstructive sleep apnea, hypoxia, galectin-3, biomarker, inflammation

## Abstract

**Background/Objectives:** Obstructive sleep apnea (OSA) is a condition characterized by intermittent airway obstructions, leading to reduced oxygen levels and increased sympathetic nervous system activity. OSA can cause a range of health problems, including an increased risk of cardiovascular diseases and mortality. Galectin-3, a member of the galectin family, plays a significant role in inflammation and fibrosis, and studies show that it is elevated in various conditions, including heart and lung diseases. The aim of this study was to determine whether galectin-3 levels are related to the severity of sleep apnea. **Methods:** A total of 191 participants from the University Clinical Hospital Center Bezanijska Kosa, Belgrade, Serbia, between January 2023 and May 2024, were included in the analyses. All patients were hospitalized under suspicion of OSA, and they all underwent a polysomnography test. Various demographic, respiratory, laboratory, and clinical parameters were obtained. Correlations between numerical variables and galectin-3 were assessed by the Pearson or Spearman correlation coefficients. Univariate and multivariate linear regression models were used to assess the predictors of galectin-3 values. In all analyses, the significance level was set at 0.05. **Results:** The mean age of the study participants was 56.2 years, mostly male (68.9%). Of the comorbidities, two-thirds of patients had hypertension (66.1%), 46.8% had hyperlipoproteinemia, and 21.1% had diabetes mellitus. Patients who had an AHI of more than fifteen events per hour more often had higher values of galectin-3. OSA severity had a significant positive correlation with galectin-3 (*p* = 0.014). In multivariate linear regression analysis, significant independent predictors of higher galectin-3 values were older age, presence of coronary disease, hypoventilation syndrome, higher BMI, NTproBNP, lactate, creatinine, lower LDL, and lower FEV1 (*p* < 0.05). **Conclusions:** The present study demonstrated that galectin-3 is linked to the severity of OSA and plays a crucial role in inflammation induced by intermittent hypoxia in OSA. Further screening and interventions targeting galectin-3 could aid in preventing inflammatory diseases related to sleep disturbances.

## 1. Introduction

Obstructive sleep apnea (OSA) is a condition defined by episodes in which the upper airway partially (hypopnea) or completely (apnea) collapses [1]. These breathing disruptions cause intermittent disturbances in blood gases, along with periods of increased sympathetic nervous system activity [2].

The severity of OSA is classified as follows, based on the Apnea–Hypopnea Index (AHI): mild (AHI = 5–14), moderate (AHI = 15–29), and severe (AHI ≥ 30). The prevalence of OSA in the general adult population in Europe is estimated to be around 44%, with approximately 23% of individuals affected by moderate to severe OSA (AHI ≥ 15) [3]. Loud snoring, unrefreshing sleep, excessive daytime sleepiness (EDS), fatigue, and observed apneas are the most commonly reported symptoms in patients with OSA. In addition, symptoms such as gastroesophageal reflux, nocturia, and chronic morning headaches are more frequently seen in OSA patients compared to the general population [4]. The disorder is associated with a wide range of metabolic disturbances and an increased risk of both all-cause mortality and cardiovascular mortality [5]. Intermittent hypoxia during sleep is the primary pathophysiological mechanism underlying the development of various diseases in patients with obstructive sleep apnea (OSA). This repeated cycle of oxygen desaturation and reoxygenation during sleep plays a crucial role in the onset and progression of comorbid conditions associated with OSA [6]. Among other things, systemic inflammation and its effects are crucial aspects in patients with OSA, while intermittent hypoxia also induces the activation of inflammatory cells and the release of inflammatory mediators [7]. One such mediator is galectin-3, a chimera-type member of the galectin family, which has emerged as a key player in cardiac inflammation and vascular processes [8].

Galectins are a family of widely expressed β-galactoside-binding lectins that play a key role in modulating both “cell-to-cell” and “cell-to-matrix” interactions across all organisms. They are categorized into three subgroups based on the number of carbohydrate recognition domains (CRDs) they contain and their functional properties: proto-type galectins (e.g., galectin-1, -2, -5, -7, -10, -11, -13, -14, and -15), tandem-repeat galectins (e.g., galectin-4, -6, -8, -9, and -12) and chimera-type galectin (e.g., galectin-3) [9]. Galectin-3, as the focus of this paper, plays a key role in inflammation and fibrosis across a range of diseases, affecting vital organs such as the heart, liver, kidneys, lungs, and brain. The primary cell types involved in galectin-3-driven disease processes include myofibroblast, epithelial cells, endothelial cells, and macrophages [10]. Galectin-3 exerts its effects by modulating various cellular compartments. It amplifies cytokine production, immune cell activation, and fibrosis cascades in a hierarchical manner, thereby influencing a broad spectrum of cardiovascular disorders [11]. Also, galectin-3 is extensively expressed in lung tissues, including bronchial and alveolae epithelial cells, the pulmonary vasculature, and immune cells such as alveolar macrophage. It has been shown to be significantly upregulated in various types of pneumonia, including bacterial, viral, and fungal pneumonia, and also in patients with chronic obstructive pulmonary disease (COPD) and COPD exacerbations. It is suggested to play a crucial role as a key regulator of the inflammatory response in these conditions [12].

In many studies, galectin-3 is described as a novel prognostic biomarker with a strong predictive value for cardiovascular mortality and hospital re-admission in heart failure (HF), conditions that are prevalent in patients with OSA [13].

There are still insufficient studies on the connection between galectin-3 and OSA, but some findings suggest elevated galectin-3 levels in women with OSA [14]. The present study aims to investigate the correlation between galectin-3 values and the severity of OSA, as well as the correlation with other important laboratory and clinical parameters.

## 2. Materials and Methods

A total of 191 participants from the University Clinical Hospital Center Bezanijska Kosa, Belgrade, Serbia, between January 2023 and May 2024, were included in the analyses. We measured galectin-3 in all patients that were enrolled in the study. The examinations were conducted in the morning following an overnight fast. The assessment included a review of medical and medication history, anthropometric measurements, glucose levels, and hemoglobin A1c. Blood pressure was measured upon hospital admission after a 5 min sitting rest using an automated device. Hypertension was defined as a history of high blood pressure, the use of antihypertensive medications, or a blood pressure reading exceeding 140/90 mmHg. All participants underwent an overnight, in-hospital sleep apnea evaluation. The sleep study was performed using the Polypro H2 series, which provides a validated estimate of the Apnea Hypopnea Index (AHI) by recording information on finger pulse oximetry, chest movement, and snoring and body position. The severity of sleep apnea was defined by using conventional clinical categories: none (AHI ≤ 5 apnea–hypopnea events per hour), mild (AHI > 5 to ≤15 apnea–hypopnea events per hour), moderate (AHI > 15 to ≤30 apnea–hypopnea events per hour), and severe (AHI > 30 apnea–hypopnea events per hour). The oxygen desaturation index (ODI) was also recorded. The ODI is the number of oxygen desaturation events (4% minimum desaturation) per estimated hour of sleep.

Biomarkers were measured at the University Clinical Hospital Center Bezanijska Kosa central laboratory. Galectin-3 was measured using a chemiluminescent microparticle immunoassay on an Alinity instrument (Abbott, Abbott Park, IL, USA) in EDTA-plasma.

Additionally, we measured high-sensitivity troponin-T (hsTnT), a biomarker for myocardial injury, as well as N-terminal pro-B-type natriuretic peptide (NT-proBNP), which indicates hemodynamic stress and neurohormonal activation. C-reactive protein (CRP), a marker of inflammatory processes, was also assessed. Arterial blood gas analysis and spirometry were conducted for all patients.

Baseline characteristics of the study population were organized by categorizing individuals according to the severity of their sleep apnea. To assess the relationship between galectin-3 and the severity of sleep apnea, Spearman correlation coefficients were used for continuous variables. Linear regression models were then applied to examine the association between galectin-3 levels and the different categories of sleep apnea severity.

### 2.1. Statistical Analysis

Numerical data were presented as mean with a 95% confidence interval, or median with 25th and 75th percentile. Categorical variables were summarized by absolute numbers with percentages. Correlations between numerical variables and galectin-3 were assessed by the Pearson or Spearman correlation coefficient. Chi-squared tests and T tests for independent samples were used to assess the differences in demographic data and comorbidities of the study population according to OSA severity. Univariate and multivariate linear regression models were used to assess predictors of galectin-3 values. In all analyses, the significance level was set at 0.05. Statistical analysis was performed using IBM SPSS statistical software (SPSS for Windows, release 25.0, SPSS, Chicago, IL, USA).

### 2.2. Institutional Review Board Statement

This study was conducted according to the guidelines of the Declaration of Helsinki and approved by the Ethics Committee of the University Hospital Medical Center Bezanijska Kosa (protocol number 41/2024).

## 3. Results

A total of 191 patients, mostly male (68.9%) and with a mean age of 56.2 years, were included in the study. Of the comorbidities, two-thirds of patients had hypertension (66.1%), 46.8% had hyperlipoproteinemia, and 21.1% had diabetes mellitus. Demographic data and comorbidities in the study population and their correlation with galectin-3 were presented in Table 1.

In Table 2, the arterial gas analysis parameters of the study population and their correlation with galectin-3 were presented. A positive correlation was found between lactate and galectin-3 values, where higher values of lactate were associated with higher values of galectin-3 (r = 0.203; *p* = 0.011). No significant correlation was observed between other examined arterial gas analysis parameters and galectin-3 (*p* > 0.05).

The clinical, lipid, and inflammation parameters of the study population and their correlation with galectin-3 are presented in Table 3. A positive correlation was found between galectin-3 values and neutrophils (r = 0.198; *p* = 0.011), urea (r = 0.554; *p* < 0.001), creatinine (r = 0.399; *p* < 0.001), LDH (r = 0.159; *p* = 0.042), troponine (rho = 0.303; *p* < 0.001), NTproBNP (rho = 0.423; *p* < 0.001), HbA1c (r = 0.247; *p* = 0.002), TSH (rho = 0.180; *p* = 0.021), fibrinogen (r = 0.195; *p* = 0.015), and microalbumine/Cr ratio (rho = 0.179; *p* = 0.025). A significant negative correlation was observed between galectin-3 values and cholesterol values (r = −0.229; *p* = 0.003), LDL (r = −0.225; *p* = 0.004), nonHDL (r = −0.252; *p* = 0.002), and albumine values (r = −0.209; *p* = 0.007).

In Table 4, the respiratory parameters of the study population and their correlation with galectin-3 are presented. Significant negative correlations between FVC, FEV1, and galectin-3 values were found, where higher values of galectin-3 were associated with lower values of FVC and FEV1 (*p* = 0.020 and *p* = 0.011, respectively). A significant positive correlation was found between galectin-3 values and ODI (rho = 0.016; *p* = 0.016).

In Table 5, OSA severity according to the demographic data and comorbidities of the study population is presented. Patients with severe OSA were older (*p* = 0.015), were more often male (*p* = 0.042), had higher neck and waist circumferences (*p* < 0.001 and *p* < 0.001, respectively), had higher BMIs (*p* < 0.001), were more often hypertensive (*p* < 0.001), had diabetes mellitus (*p* = 0.039), and more often had hypoventilation syndrome (*p* = 0.011).

**Figure 1:**
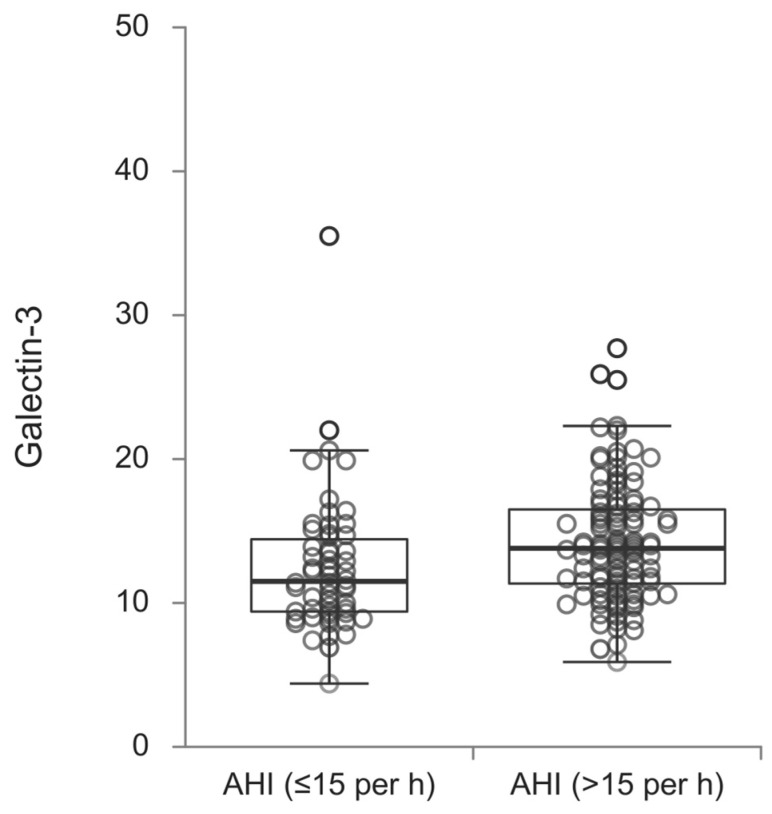
Galectin-3 values according to the OSA severity.

The galectin-3 values significantly differed according to the examined groups (*p* = 0.014), where patients who had an AHI of more than fifteen events per hour more often had higher values of galectin-3 (Figure 1).

In Table 6, univariate linear regression analysis with galectin-3 as a dependent variable is presented. Out of the demographic data and comorbidities of the study population, the significant predictors of galectin-3 were as follows: older age (*p* < 0.001), waist circumference (*p* = 0.003), BMI (*p* = 0.015), smoking (*p* = 0.032), presence of hypertension (*p* < 0.001), diabetes mellitus (*p* = 0.005), hypoventilation syndrome (*p* < 0.001), coronary disease (*p* < 0.001), and cardiomyopathy (*p* < 0.001). Higher values of lactate (*p* = 0.011), neutrophils (*p* = 0.011), urea (*p* < 0.001), creatinine (*p* < 0.001), direct bilirubin (*p* = 0.023), LDH (*p* = 0.042), GGT (*p* = 0.012), troponine (*p* = 0.032), NTproBNP (*p* < 0.001), HbA1c (*p* = 0.002), and fibrinogen (*p* = 0.015), as well as lower values of cholesterol (*p* = 0.003), albumine (*p* = 0.007), LDL (*p* = 0.004), and nonLDL (*p* = 0.002), were significant predictors of higher values of galectin-3. Out of the respiratory parameters of the study population, the significant predictors of higher values of galectin-3 were lower FVC (*p* = 0.020) and FEV1 (*p* = 0.011). OSA severity had a significant positive correlation with galectin-3 (*p* = 0.014) (Table 6).

Multivariate linear regression analysis with galectin-3 as a dependent variable is presented in Table 7. The significant independent predictors of higher galectin-3 values were as follows: older age (*p* < 0.001), presence of coronary disease (*p* < 0.001), hypoventilation syndrome (*p* = 0.025), higher BMI (*p* = 0.034), NTproBNP (*p* < 0.001), lactate (*p* = 0.005), creatinine (*p* = 0.009), lower LDL (*p* = 0.023), and lower FEV1 (*p* = 0.011).

## 4. Discussion

In the present study, where we assessed the role of galectin-3 in predicting the severity of OSA, galectin-3 levels were significantly elevated in patients with severe sleep apnea. The patients with a higher AHI were older, predominantly male, had higher BMIs, waist and neck circumferences, and had hypertension and diabetes mellitus more often. The significant independent predictors of higher galectin-3 values were older age, presence of coronary disease, hypoventilation syndrome, higher BMI, NTproBNP, lactate, creatinine, lower LDL, and lower FEV1.

Systemic inflammation and its impact are critical factors in OSA patients, as intermittent hypoxia triggers the activation of inflammatory cells and the release of inflammatory mediators [7]. Furthermore, galectin-3 plays a key role in inflammation and fibrosis across various diseases, affecting vital organs such as the heart, lungs, brain, liver, and kidneys.

Pusuroglu et al. also investigated the relationship between OSA severity and galectin-3 levels [15]. Galectin-3 was associated with OSA severity, but also with coronary atherosclerosis estimated by coronary CT angiography.

Among the demographic data and comorbidities of the study population, significant predictors included older age, waist circumference, body mass index (BMI), smoking, hypertension, diabetes mellitus, hypoventilation syndrome, coronary artery disease, and cardiomyopathy. A study by Singh et al. demonstrated that sleep apnea is associated with elevated galectin-3 levels in women but not men [14]. Considering that the patients with a higher AHI in our study had a higher BMI and had hypertension and diabetes mellitus more often, the potential correlation of galectin-3 with low-grade systemic inflammation should also be taken into account.

A major finding of our study is the strong association between elevated galectin-3 levels and traditional metabolic biomarkers. This result supports the existing literature that highlights galectin-3’s important role in the pathophysiology of cardiometabolic diseases [16]. Galectin-3 was found to be positively correlated with neutrophil count, urea, creatinine, troponin, NT-proBNP, LDH, HbA1c, TSH, fibrinogen, and the microalbumin/creatinine ratio, while showing a negative correlation with cholesterol levels, LDL, non-HDL, and albumin levels.

Tan et al. [17] reported that diabetic patients with complications, who had significantly poorer glycemic control (with an HbA1c of 9.45 ± 2.31% as a reference), exhibited markedly higher serum galectin-3 levels compared to diabetic patients without complications (*p* < 0.001). Similarly, studies by Weigert et al. [18], Jin Qi-hui et al. [19], and Hodeib et al. [20] also found a positive correlation between plasma galectin-3 levels and HbA1c levels.

Galectin-3 enhances the adhesion of human neutrophils [21,22]. Furthermore, in an in vivo mouse model of streptococcal pneumonia, neutrophil extravasation was closely linked to the accumulation of galectin-3 in the alveolar space, a process that occurred independently of β2-integrin [22].

In our study, a negative correlation was found between galectin-3 levels and both FVC and FEV1, confirming that galectin-3 levels are higher during exacerbations of COPD and asthma, which are characterized by lower values of FEV1 and FVC. Two studies have shown that galectin-3 levels are elevated in both the blood and lung tissues of COPD patients [23,24]. Consistent with these findings, it is suggested that galectin-3 may serve as a valuable marker for the early detection of exacerbations [25].

Galectin-3 is involved in various crucial mechanisms of asthma pathophysiology, such as the allergic response, eosinophil activation, and non-Th2 inflammation. As a result, galectin-3 holds potential as a valuable biomarker for diagnosing and monitoring asthma over time [26].

NT-proBNP, released by cardiomyocytes in response to ventricular strain, is a marker of right ventricular dysfunction [25]. When combined with BNP, galectin-3 improves predictive accuracy in patients discharged after an acute decompensated heart failure episode, offering better prognostic value than BNP alone [27]. In our study, we found a significant positive correlation between galectin-3 and NT-proBNP levels. Increased galectin-3 levels may be associated with higher pulmonary vascular resistance due to hypoxia, oxidative stress, and both systemic and pulmonary inflammation.

Berber, NK et al. reported a negative correlation between serum galectin-3 levels and PaO2 levels [25]. In our study, we observed a positive correlation between lactate and galectin-3 levels, but no significant correlation was found between galectin-3 and other examined arterial blood gas parameters.

Albumin, an acute-phase negative protein, also has antioxidant properties. During the acute-phase response, circulating albumin levels generally decrease [28]. In line with this, our study found a negative correlation between galectin-3 levels and albumin levels. This suggests that an increased systemic inflammatory response and oxidative stress may contribute to elevated galectin-3 concentrations and reduced albumin levels in OSA patients.

The repetitive cycles of hypoxia and reoxygenation during sleep in OSA contribute to oxidative stress, driven by the increased production of reactive oxygen species, angiogenesis, and enhanced sympathetic activation [29]. Galectin-3 has been identified as a critical regulator of oxidative stress, and its inhibition has been shown to restore the antioxidant peroxiredoxin-4, thereby alleviating oxidative stress [30].

However, research on the role of galectin-3 in obstructive sleep apnea is still insufficient. Future studies will shed light on the importance of this biomarker for the prognosis of patients with OSA.

The present study has several limitations. This was a single-center and non-randomized study, and therefore, was subject to selection bias. Also, the results may not be applicable to other ethnic groups or populations. Bearing in mind the role of galectin-3 in myocardial fibrosis, future analysis of cardiovascular risk in patients with severe OSA should be considered. Further research is needed to evaluate the usefulness of galectin-3 as a parameter in monitoring patients after the initiation of therapy, as well as to analyze its association with outcomes such as hospitalization rates, cardiovascular complications, and mortality.

## 5. Conclusions

Considering that galectin-3 is linked to the severity of OSA and plays a crucial role in inflammation induced by intermittent hypoxia in OSA, further screening and interventions targeting galectin-3 could aid in preventing inflammatory diseases related to sleep disturbances.

## Figures and Tables

**Table 1:** Demographic data and comorbidities of the study population and their correlation with galectin-3.

Variable	Total (n = 191)	Galectin-3 r
Age, mean (95%CI), years	56.2 (54.3–58.1)	0.423 **
Gender, male	131 (68.9)	0.122
Smoking	16 (8.4)	0.165 *
Neck circumference, mean (95%CI), cm	42.5 (41.6–43.4)	0.042
Waist circumference, mean (95%CI), cm	110.4 (107.8–113.0)	0.238 *
BMI, mean (95%CI), cm	32.5 (31.5–33.7)	0.193 *
Hypertension	125 (66.1)	0.329 **
Diabetes mellitus	40 (21.1)	0.213 **
COPD	14 (7.4)	0.029
Hypoventilation syndrome	12 (6.3)	0.294 **
Asthma	15 (7.9)	0.083
Coronary disease	22 (11.6)	0.341 **
Cardiomyopathy	19 (10.0)	0.351 **
Hypothyroidism	19 (10.0)	0.099
Hyperlipoproteinemia	88 (46.8)	−0.063
Malignancy	4 (2.1)	−0.011

BMI, body mass index; COPD, chronic obstructive pulmonary disease. * *p* < 0.05; ** *p* < 0.01.

**Table 2:** Arterial gas analysis parameters of the study population and their correlation with galectin-3.

Variable	Total (n = 191)	Galectin-3 r
pH, mean (95%CI)	7.43 (7.42–7.43)	0.039
pCO2, mean (95%CI)	5.37 (5.27–5.47)	−0.107
pO2, mean (95%CI)	11.06 (10.74–11.38)	−0.098
sO2, mean (95%CI)	96.20 (95.77–96.62)	−0.125
HCO3, mean (95%CI)	26.89 (26.47–27.32)	−0.084
Lactate, mean (95%CI)	1.12 (1.03–1.21)	0.203 *

* *p* < 0.05.

**Table 3:** Clinical, lipid, and inflammation parameters of the study population and their correlation with galectin-3.

Variable Mean (95%CI)	Total (n = 191)	Galectin-3 r/rho
Erythrocytes	4.73 (6.64–4.82)	−0.019
Leukocytes	7.34 (6.86–7.83)	0.141
Neutrophils	59.56 (57.98–61.13)	0.198 *
Lymphocytes	2.20 (1.93–2.48)	−0.085
Thrombocytes	224.6 (215.77–233.43)	−0.048
Hemoglobin	141.35 (138.49–144.20)	−0.135
Urea	6.17 (5.77–6.57)	0.554 **
Creatinine	86.98 (82.55–91.40)	0.399 **
Glycose	6.35 (5.88–6.82)	0.040
Total bilirubin	2.71 (2.48–2.96)	0.174 *
Direct bilirubin	(11.51 (10.43–12.60)	0.114
AST	21.62 (20.02–23.22)	0.097
ALT	26.33 (23.14–29.51)	−0.065
LDH	369.4 (351.63–387.17)	0.159 *
Triglycerides	2.03 (1.82–2.25)	−0.091
Cholesterol	4.82 (4.59–5.04)	−0.229 **
HDL	1.14 (1.09–1.20)	−0.053
LDL	2.83 (2.63–3.04)	−0.225 **
nonHDL	3.72 (3.50–3.94)	−0.252 **
CK, median (25–75th percentile)	110.0 (81.0–174.0)	−0.071
Troponine, median (25–75th percentile)	5.0 (3.0–9.0)	0.303 *
NTproBNP, median (25–75th percentile)	74.64 (35.0–158.0)	0.423 **
GGT, median (25–75th percentile)	23.0 (15.0–34.0)	−0.030
Albumine	45.05 (44.45–45.65)	−0.209 *
CRP, median (25–75th percentile)	1.90 (0.90–5.70)	0.142
HbA1c	5.94 (5.71–6.17)	0.247 **
FT4	15.05 (14.59–15.52)	0.093
TSH, median (25–75th percentile)	1.78 (1.14–2.58)	0.180 *
Fibrinogen	3.21 (3.07–3.35)	0.195 *
Microalbumine/Cr ratio, median (25–75th percentile)	0.80 (0.45–1.97)	0.179 *

* *p* < 0.05; ** *p* < 0.01.

**Table 4:** Respiratory parameters of the study population and their correlation with galectin-3.

Variable Mean (95%CI)	Total (n = 191)	Galectin-3 r/rho
FVC, mean (95%CI)	91.69 (88.92–94.48)	−0.183 *
FEV1, mean (95%CI)	92.77 (89.87–95.66)	−0.200 *
FVC % FEV1, mean (95%CI)	81.28 (80.20–82.36)	−0.110
ODI, median (25–75th percentile)	24.15 (8.90–50.20)	0.186 *

* *p* < 0.05.

**Table 5:** OSA severity according to demographic data and comorbidities of the study population.

	OSA Severity		*p*
	AHI (≤15 per h)	AHI (>15 per h)	
Age, mean (95%CI), years	52.6 (50.0–56.2)	57.7 (55.7–59.8)	0.015
Gender, male	38 (59.4)	93 (73.8)	0.042
Smoking	4 (6.3)	12 (9.5)	0.442
Neck circumference, mean (95%CI), cm	39.6 (38.5–40.7)	43.9 (42.8–45.0)	<0.001
Waist circumference, mean (95%CI), cm	99.1 (95.7–102.5)	115.7 (112.6–118.7)	<0.001
BMI, mean (95%CI), cm	28.6 (27.1–30.2)	34.5 (33.2–35.8)	<0.001
Hypertension	31 (48.4)	94 (75.2)	<0.001
Diabetes mellitus	8 (12.5)	32 (25.4)	0.039
COPD	2 (3.1)	12 (9.6)	0.108
Hypoventilation syndrome	0 (0.0)	12 (9.5)	0.011
Asthma	6 (9.5)	9 (7.1)	0.568
Coronary disease	7 (10.9)	15 (11.9)	0.844
Cardiomyopathy	5 (7.8)	14 (11.1)	0.474
Hypothyroidism	6 (9.4)	13 (10.3)	0.838
Hyperlipoproteinemia	30 (47.6)	58 (46.4)	0.874
Malignancy	0 (0.0)	4 (3.2)	0.150

**Table 6:** Univariate linear regression analysis with galectin-3 as dependent variable.

Variable	β	t	*p*
Age	0.423	6.037	<0.001
Waist circumference	0.238	3.025	0.003
BMI	0.193	2.459	0.015
Smoking	0.165	2.160	0.032
Hypertension	0.329	4.483	<0.001
Diabetes Mellitus	0.213	2.823	0.005
Hypoventilation syndrome	0.294	3.959	<0.001
Coronary disease	0.341	4.684	<0.001
Cardiomyopathy	0.351	4.848	<0.001
Lactate	0.203	2.573	0.011
Neutrophils	0.198	2.587	0.011
Urea	0.554	8.595	<0.001
Creatinine	0.399	5.626	<0.001
Direct bilirubin	0.174	2.289	0.023
LDH	0.159	2.047	0.042
GGT	0.196	2.544	0.012
Cholesterol	−0.229	−3.000	0.003
Albumine	−0.209	−2.749	0.007
LDL	−0.225	−2.937	0.004
nonLDL	−0.252	−3.211	0.002
Troponine	0.174	2.158	0.032
NTproBNP	0.373	5.124	<0.001
HbA1c	0.247	3.147	0.002
Fibrinogen	0.195	2.472	0.015
FVC	−0.183	−2.355	0.020
FEV1	−0.200	−2.577	0.011
OSA severity	0.188	2.480	0.014

**Table 7:** Multivariate linear regression analysis with galectin-3 as dependent variable.

Variable	β	t	*p*
Age	0.394	5.809	<0.001
Coronary disease	0.306	4.470	<0.001
Hypoventilation syndrome	0.157	2.266	0.025
BMI	0.147	2.136	0.034
NTproBNP	0.391	4.793	<0.001
Lactate	0.244	2.891	0.005
Creatinine	0.216	2.654	0.009
LDL	−0.194	−2.312	0.023
FEV1	−0.200	−2.577	0.011

## Data Availability

The data that support the findings of this study are available from the corresponding author (M.B.) upon reasonable request.
